# Dynamic transcriptome analysis highlights collagen-integrin-mediated extracellular matrix remodeling underlying sexual dimorphism in the amplexus muscle of *Bufo gargarizans*

**DOI:** 10.1186/s13293-026-00851-7

**Published:** 2026-02-08

**Authors:** Zhiping Mi, Xinyu Liu, Yanmei Liu, Hui Ma, Chengzhi Yan

**Affiliations:** 1 Key Laboratory of Southwest China Wildlife Resources Conservation (Ministry of Education), China West Normal University, Nanchong, Sichuan China; 2 College of Life Science, China West Normal University, Nanchong, Sichuan China

**Keywords:** Sexual dimorphism, Muscle development, Extracellular matrix, Amplexus, Anurans

## Abstract

**Background:**

Sexual dimorphism in skeletal muscle is a well-recognized biological phenomenon of adaptive evolution, yet its developmental genetic basis remains poorly understood. In many anurans, males develop hypertrophied forelimb muscles to facilitate amplexus (mating embrace) during breeding season, offering an ideal model to dissect the ontogeny of sex-specific muscle growth. The aim of this study was to determine the developmental onset of sexual dimorphism in the flexor carpi radialis (FCR) muscle and to characterize the transcriptional landscape driving this phenotypic divergence.

**Methods:**

We first established the developmental timeline of sexual dimorphism in the FCRmuscle of *Bufo gargarizans* through morphological analysis. Based on the identified onset time, we performed comparative RNA sequencing at three critical post-metamorphic stages: pre-dimorphism (PD; 2 months post-metamorphosis), onset of dimorphism (OD; 4 months post-metamorphosis), and adult (AD; 24 months post-metamorphosis), to capture transcriptional dynamics associated with phenotype emergence.

**Results:**

The results showed that males exhibited significantly greater FCR mass than females beginning at the OD stage and this difference persisted into adulthood. Transcriptomic profiling revealed a marked increase in sex‑biased differentially expressed genes (DEGs) at OD, coinciding with the emergence of morphological divergence. Time‑series analysis identified two major expression trajectories: 415 genes showed a sharp increase in male-biased expression from PD to OD, followed by a decline in AD, whereas 177 genes increased continuously from PD through AD. Integrated analysis highlighted eight candidate genes, comprising seven collagen isoforms (*COL1A1*, *COL2A1*, *COL4A1*, *COL6A1*, *COL6A2*, *COL6A3*, *COL6A6*) and an integrin β subunit (*ITGB6*), which showed coordinated male‑biased expression at OD stage. Protein-protein interaction network predicted ITGB6 as a critical hub linking extracellular matrix (ECM) remodeling to intracellular focal adhesion and FAK signaling pathways.

**Conclusions:**

These findings support a developmental model in which collagen-integrin-mediated ECM remodeling at a critical post-metamorphic stage, well before sexual maturation. This process establishes and stabilizes a sexually dimorphic muscle phenotype optimized for the mechanical demands of amplexus.

**Highlights:**

Male-biased hypertrophy of the flexor carpi radialis (FCR) muscle in male *B. gargarizans* emerges within a specific post-metamorphic pre-maturation window, accompanied by a surge in male-biased transcription.

A sex-biased co-expressed gene set comprising seven collagen isoforms and the integrin subunit ITGB6 shows coordinated upregulation specifically at the onset of sexually dimorphic muscle development.

Network analysis positions ITGB6 as a mechanotransduction hub that links extracellular collagen matrix remodeling (ECM) to intracellular focal adhesion and FAK signaling pathways, providing a molecular basis for sex-specific muscle growth.

**Plain Language Summary:**

In many animals, males and females differ in their physical traits known as sexual dimorphism. In frogs and toads, for example, males often have stronger forelimb muscles that help them clasp females during mating. However, it was unclear when these muscle differences first appear during development and what genetic instructions make them grow differently. We investigated these questions in Asiatic toads (B. gargarizans). Our study clarifies that males begin to exhibit significant hypertrophy of the flexor carpi radialis muscle at four months after metamorphosis, well before sexual maturation. We found that this growth is accompanied by an increase in the activity of specific genes. We identified eight candidate genes involved in extracellular matrix remodeling—a process that reorganizes the muscle microenvironment and influences its architecture. Among these, a protein called ITGB6 acts as a molecular bridge, connecting the extracellular matrix to internal signaling pathways within muscle cells that help regulate muscle growth. Our study highlights that sex-biased transcriptional changes during early development coincide with lasting differences between males and females, providing insight into the potential biological basis of sexually dimorphic traits in vertebrates

**Supplementary Information:**

The online version contains supplementary material available at 10.1186/s13293-026-00851-7.

## Background

Sexual dimorphism is a widespread and evolutionarily significant phenomenon in animals, encompassing differences in body size, coloration, morphology, physiological traits, and behavior between males and females of the same species [1–3]. These sex-specific characteristics are shaped by a complex interplay of evolutionary forces, including natural selection, sexual selection, and fecundity selection [4]. Sexual dimorphism not only influences reproductive success but also affects resource allocation, social structure, and ecological niche partitioning within populations [5–7]. Consequently, these differences contribute to individual fitness as well as to population-level structure and dynamics. While previous researches have predominantly focused on describing dimorphic traits and their adaptive significance [8–10], the developmental timing and underlying molecular mechanisms driving sexual dimorphism remain insufficiently understood.

Among sexually dimorphic traits, skeletal muscle specialization is particularly notable, especially when linked to reproductive behaviors. The functional capacity of these muscles is often tightly coupled with mating strategies and is frequently subject to strong sexual selection. Such adaptations have been documented across diverse vertebrate lineages. For example, in certain birds, males have evolved more robust laryngeal muscles to produce complex courtship calls [10, 11], while in some fish, males possess enlarged sonic muscles that facilitate acoustic signaling for mate attraction and territorial defense [12, 13]. Similarly, in most anurans, forelimb muscles involved in amplexus are more developed in males, enabling them to securely grasp females during mating and ensure successful external fertilization [14]. These sexual differences in muscle mass are associated with smaller fiber diameters, lower fiber numbers, and reduced protein content in female forelimb muscles [15, 16]. Physiologically, male forelimb muscles also exhibit greater absolute force, longer contraction-relaxation cycles, and enhanced fatigue resistance, reflecting functional adaptations that enhance mating efficiency during the breeding season [17, 18].

Sexual dimorphism in skeletal muscle reflects the combined forces of evolutionary selective pressures, mediated by an intricate interplay of hormonal and genetic factors [4]. Sex hormones, particularly androgens, play a central role in promoting protein synthesis, muscle fiber hypertrophy, and functional differentiation [19]. For example, in *Rana catesbeiana*, male laryngeal muscles exhibit high androgen receptor abundance, correlating with larger muscle cross-sectional areas and a predominance of fast-twitch fibers essential for producing advertisement calls [20]. Beyond hormonal control, sex chromosomes exert intrinsic effects on muscle traits. Evidence from the Four-Core Genotypes (FCG) mouse model, which separates chromosomal from gonadal sex, indicates that the sex chromosome complement (XX vs. XY) directly influences muscle mass and metabolism independent of hormonal signaling [21, 22]. At the molecular level, these genetic and hormonal signals converge at the transcriptional regulation. According to the “sexually dimorphic genetic switch” model, cis-regulatory elements (CREs) act as integration nodes, interpreting and assembling inputs from both sex-specific transcription factors (e.g., hormone-receptor complexes) and tissue-specific regulators, thereby directing the spatiotemporal dynamics of downstream gene networks [23]. However, these insights are predominantly derived from analyses of specific candidate genes and pathways. Consequently, a comprehensive map of the gene networks dynamically activated during development to establish and maintain skeletal muscle sexual dimorphism remains insufficiently understood.

The Asiatic toad (*Bufo gargarizans*) serves as an ideal for investigating skeletal muscle sexual dimorphism due to its pronounced, multi-level differences between the sexes. Adult males are smaller than females but possess specialized traits adapted for reproductive competition, including keratinized nuptial pads and significantly more robust forelimb skeletons characterized by heavier humeri and radioulnas with enlarged crest areas [24, 25]. These morphological adaptations are paralleled by physiological divergence: females tend to maintain larger digestive organs and store more lipids in visceral fat bodies to meet the energetic costs of gametogenesis, whereas males display sex-specific metabolic characteristics in adipose tissues that may help preserve gonadal function under oxidative stress [26, 27]. Among these traits, our previous work demonstrated that the flexor carpi radialis (FCR) muscle, which functions in amplexus during the breeding period, exhibits marked sexual dimorphism in mass [14, 28]. However, the developmental timeline and regulatory mechanisms underlying this dimorphism remain poorly characterized. Here, we integrate morphological and transcriptomic analyses across a post-metamorphic timeline to pinpoint the developmental origin of FCR muscle dimorphism and identify the key regulatory programs that trigger this divergence. Our findings provide insight into the genetic basis of sexually dimorphic traits in the FCR muscle and enhance understanding of the molecular mechanisms of muscle adaptive evolution in amphibians.

## Methods

### Sample Preparation and measurement

Asiatic toads (*B. gargarizans*) were collected from the Tuanshanba Frog Breeding Farm in Ziyang City, Sichuan Province, China, in April-September 2024. These individuals were maintained in outdoor enclosures under semi-natural conditions (ambient temperature and natural photoperiod) with standardized feeding and husbandry to minimize variation in growth. Juveniles (2, 3, 4, and 5 months post-metamorphosis) were followed longitudinally from a single current-year cohort, whereas adults (24 months post-metamorphosis) were sampled from a separate cohort with ages verified using breeding records. Here, post-metamorphic refers to the developmental period beginning immediately after completion of metamorphosis, operationally defined as complete tail resorption, marking the transition from the aquatic larval form to terrestrial juvenile and adult forms [29]. Toads were anesthetized by immersion in a buffered 0.1% MS-222 (Tricaine methanesulfonate) solution, assessed by the loss of righting reflex, and then euthanized via double pithing. The snout-vent length (SVL) of each toad was measured to the nearest 0.1 mm using precision calipers. Both flexor carpi radialis (FCR) muscles were then quickly dissected for subsequent processing. The left FCR muscle was used for mass measurement: wet mass was recorded with an analytical balance, and the muscle was then dried at 60 ℃ for 48 h to constant weight to obtain its dry mass. The right FCR was snap‑frozen and maintained in a liquid nitrogen tank until transcriptomic analysis. All experimental procedures were performed in accordance with the guidelines of the Institutional Animal Care and Use Committee of China West Normal University (Approval No. CWNU2021D006).

### RNA extraction and sequencing

Based on the morphological analysis of muscle mass, samples from three key developmental stages were selected for transcriptome sequencing: pre‑dimorphism (PD; 2 months post-metamorphosis), onset of dimorphism (OD; 4 months post-metamorphosis), and adult (AD; 24 months post-metamorphosis). Each sex at each stage included six biological replicates. Total RNA was extracted from FCR muscle tissue using TRIzol reagent (Invitrogen, USA). RNA concentration and purity were measured with a NanoDrop spectrophotometer, and integrity was verified using an Agilent 2100 Bioanalyzer. Only samples with an RNA Integrity Number (RIN) ≥ 6.0 and a 28 S/18S ratio ≥ 0.7 were retained for library construction. Sequencing libraries were prepared by OE Biotech Co., Ltd. (Shanghai, China) using the VAHTS Universal V6 RNA-seq Library Prep Kit and sequenced on an Illumina NovaSeq 6000 platform to generate 150-bp paired-end reads. Detailed procedures followed the method described in our previous study [14].

Raw sequencing reads were processed with fastp to remove adapters and low-quality sequences, generating clean reads [30]. Clean reads were aligned to the *Bufo gargarizans* reference genome using HISAT2 [31, 32]. Gene‑level read counts were obtained with HTSeq‑count for subsequent analyses, and expression levels were quantified as fragments per kilobase of transcript per million mapped reads (FPKM) [33]. Principal Component Analysis (PCA) was performed on the expression profiles of all samples to assess global transcriptomic variation. Differential expression analyses between males and females at PD, OD, and AD stages were conducted using the DESeq2 package in R (v 3.2.0) [34]. Genes were identified as differentially expressed (DEGs) if they met the criteria of an adjusted *p*-value (*q*-value) < 0.05 and |log₂ (fold change)| ≥ 1.

### Gene expression pattern clustering and functional enrichment analysis

To investigate expression tendencies of DEGs across developmental stages, the combined set of all DEGs identified from the three pairwise comparisons was analyzed using the Short Time-series Expression Miner (STEM) software. Only profiles exhibiting statistically significant temporal trends (*p* < 0.05) were retained. For downstream analyses, we focused on genes from Profile 7, exhibiting male‑biased expression at the OD stage, and Profile 8, showing sustained male‑biased expression initiated at OD, as these patterns align with the onset and maintenance of muscle dimorphism. Functional annotation was performed by aligning genes to the KEGG database to assign species-independent KEGG Orthology (KO) identifiers. Gene Ontology (GO) and Kyoto Encyclopedia of Genes and Genomes (KEGG) pathway enrichment analyses were then performed on this gene subset in R (v3.2.0). Significantly enriched terms were defined by a threshold of *p* < 0.05.

### qRT‑PCR validation of gene expression

To validate the transcriptome sequencing results, four candidate DEGs (*ASB12*, *PTX4*, *IQCA1*, and *XPO4*) were selected for quantitative real‑time PCR (qRT‑PCR) analysis. First‑strand cDNA was synthesized from the original RNA samples using the PrimeScript RT Reagent Kit (RR047A, TaKaRa, China). qRT‑PCR was conducted on a QuantStudio 3 Real‑Time PCR System (Thermo Fisher, CA, USA) with TB Green Premix Ex Taq II (RR820A, TaKaRa, China). All measurements were performed with four biological replicates, each comprising three technical replicates. Relative expression levels were calculated using the 2^(–ΔΔCt) method, with normalization to the endogenous control GAPDH. Primer sequences are listed in Table 1.

**Table 1:** List of primers used in this study

Genes	Forward Primer Sequence	Reverse Primer Sequence
*GAPDH*	CTTGAAGGTTATCAGCAATGC	CTCCACAATACCAAAGTTGTC
*ASB12*	CAGATGTCTGAAGATGAGTGT	TAACTGCCATATGTAGTTCACG
*PTX4*	CATCTTAACCATTGCTGATGC	TGGAAGTCTACAGACAGTGC
*IQCA1*	TAAGAGACACTATACGAGAGACAG	TTCTATGAACCATTGCCGAATC
*XPO4*	AGAGTTGGTGGAAACACTAT	CTTGCCGTCAGTTTGTTGAA

### Gene set enrichment analysis (GSEA) and core pathway identification

Gene Set Enrichment Analysis (GSEA) was performed with the GSEA software (v4.3.2). For each of the three comparisons (male vs. female at PD, OD, and AD stages), a complete gene list was pre-ranked according to log₂(Fold Change) values. The analysis was conducted against KEGG pathway gene sets using 1,000 permutations. Pathways were considered significantly enriched if they satisfied all of the following criteria: |NES| > 1, nominal *p* < 0.05, and FDR q < 0.05. Gene sets containing fewer than 15 genes or more than 500 genes were excluded to reduce potential bias from extreme set sizes. To identify high‑confidence core pathways, significantly enriched pathways from GSEA were intersected with the top 10 KEGG pathways obtained from conventional enrichment analysis at each developmental stage. All DEGs present in these intersecting pathways were designated as key candidate genes.

### Protein-protein interaction (PPI) network construction

Functional associations among core DEGs were assessed by first converting the *B. gargarizans* genes to their corresponding human orthologs using Ensembl BioMart. The resulting ortholog list was then submitted to the STRING database (v12, https://string-db.org) to construct the PPI network. Given the limited functional annotation for *B. gargarizans*, the analysis was performed against the *Homo sapiens* reference dataset to leverage its comprehensive homologous protein interaction data. The analysis parameters were configured to a high confidence level (minimum required interaction score > 0.700), and only interactions supported by experimental evidence and database annotations were included. The human reference was used exclusively for PPI construction and did not affect upstream read mapping or quantification, which relied on the *B. gargarizans* genome. The resulting network data from STRING was then imported into Cytoscape software (v3.10.4) for visualization and topological analysis. In this network, each node denotes a protein and edges correspond to experimentally supported or annotated interactions. The NetworkAnalyzer plugin within Cytoscape was used to calculate the degree of each node, which represents the number of direct interactions a protein has, to identify potential hub proteins.

### Statistical analyses

All statistical analyses were performed using SPSS (v25.0), with significance defined as *p* < 0.05. Data are presented as mean ± standard error (SE). To assess sex differences in muscle mass independent of body size, we used analysis of covariance (ANCOVA), with muscle mass as the dependent variable, sex as the independent variable, and snout–vent length (SVL) as the covariate.

## Results

### Developmental onset of sexual dimorphism in FCR mass

**Fig. 1:**
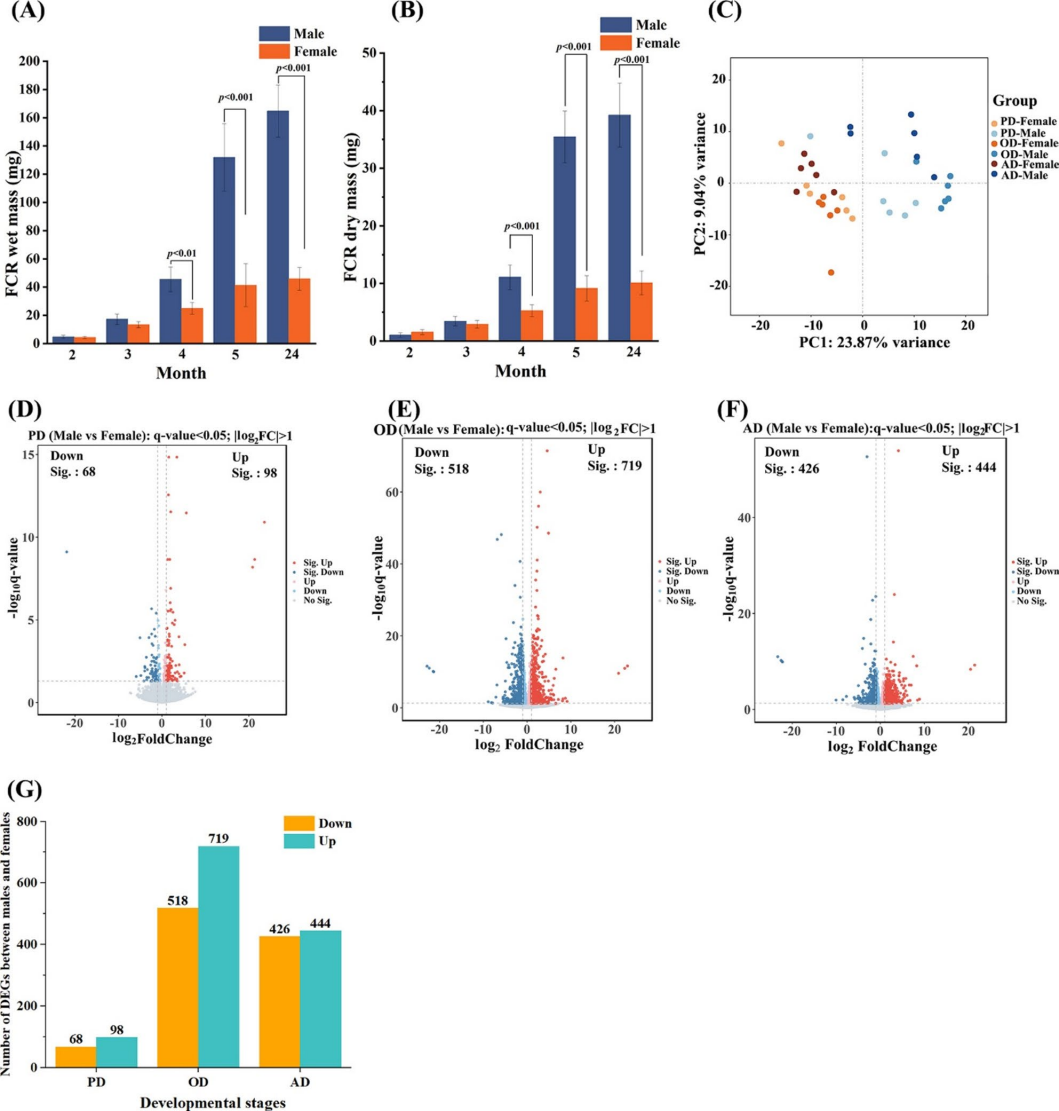
**(A-B)** Comparisons of wet mass and dry mass of the flexor carpi radialis (FCR) muscle between males and females at five post-metamorphic time points. Each bar represents the mean ± SE for each sex across developmental stages. (*n* = 6 biological replicates per sex at each developmental stage). **(C)** Principal component analysis (PCA) of transcriptomic data from male and female FCR muscles at pre-dimorphism (PD), onset of dimorphism (OD), and adult (AD) stages. **(D–F)** Volcano plots of differentially expressed genes (DEGs) between males and females at the PD, OD, and AD stages. Red and blue dots indicate significantly upregulated and downregulated genes in males relative to females (|log₂FC| ≥ 1, q < 0.05). (*n* = 6 biological replicates per sex at each developmental stage). **(G)** The number of DEGs between sexes at each developmental stage

To characterize the developmental emergence of sexual dimorphism in the flexor carpi radialis (FCR) muscle of *B. gargarizans*, we collected male and female specimens at five post-metamorphic time points (2, 3, 4, and 5 months, and adulthood at 24 months) and measured both wet and dry muscle mass. As shown in Fig. 1A and B, males exhibited significantly greater FCR wet and dry mass than females from the 4-month stage onward, and this dimorphism persisted into adulthood. These findings indicate that sexual dimorphism in the FCR muscle mass emerges before sexual maturation, with early divergence potentially serving as a preparatory adaptation to enhance forelimb grasping strength during amplexus, likely driven by sex-specific regulatory mechanisms.

### Sex-biased gene expression divergence across developmental stages

To investigate the transcriptomic basis of sexual dimorphism in the FCR muscle during development, we grouped ontogenetic process into three stages: pre-dimorphism (PD; 2 months post-metamorphosis), onset of dimorphism (OD; 4 months post-metamorphosis), and adult (AD; 24 months post-metamorphosis). Male and female samples from each stage were subjected to RNA sequencing. Principal component analysis (PCA) revealed distinct clustering patterns according to sex and developmental stage, with the greatest separation between males and females at the OD stage (Fig. 1C). Differential expression analysis (|log₂FC| ≥ 1; q < 0.05) identified 166 differentially expressed genes (DEGs) at the PD stage (98 upregulated, 68 downregulated in males relative to females), 1237 DEGs at the OD stage (719 upregulated, 518 downregulated), and 870 DEGs at the AD stage (444 upregulated, 426 downregulated) (Fig. 1D–F, Table S1-S3). As summarized in Fig. 1G, the OD stage exhibited the highest number of DEGs, underscoring this period as a critical window for the establishment of sex-specific transcriptional divergence in the FCR muscle, which coincides with the onset of mass dimorphism.

### Dynamic expression patterns of DEGs

**Fig. 2:**
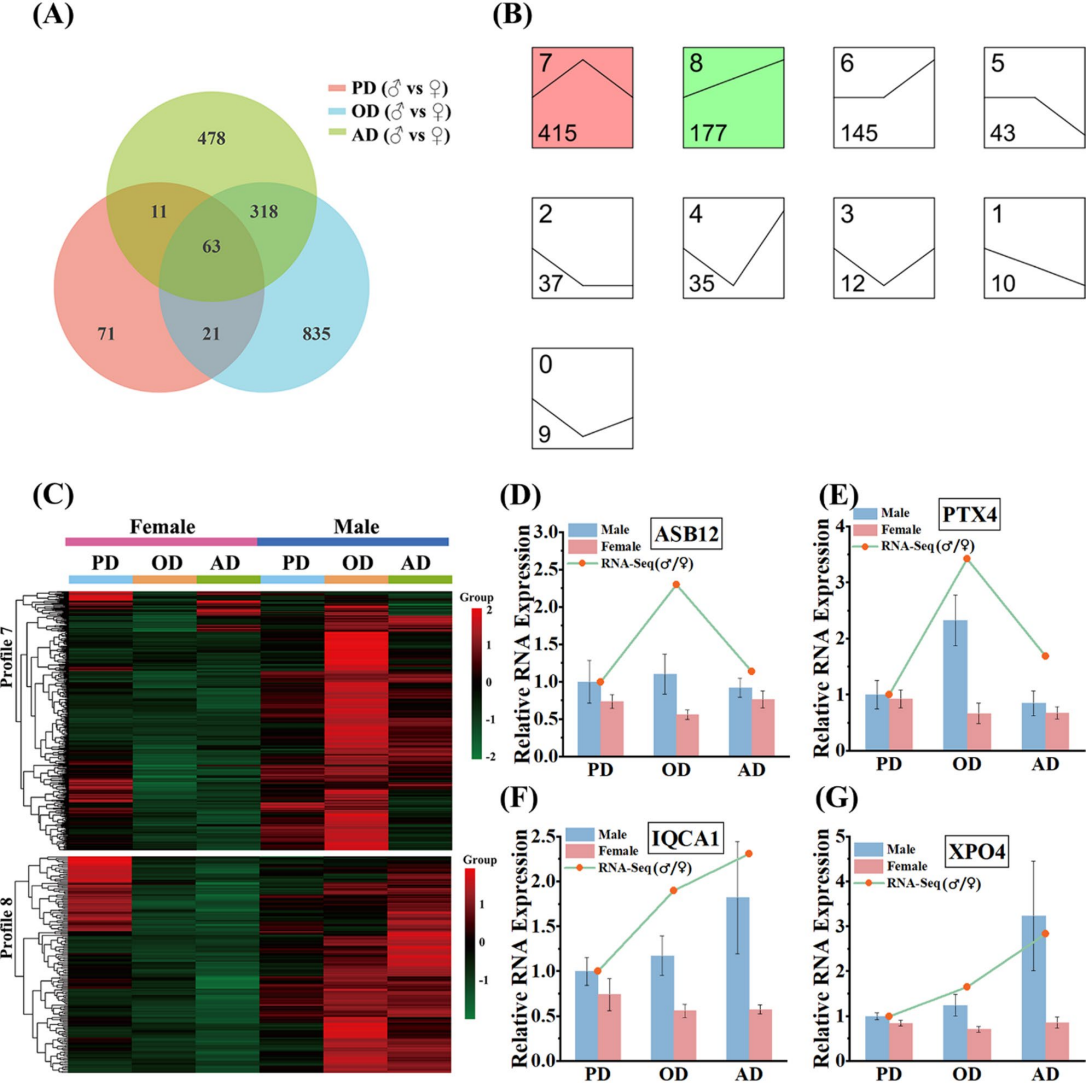
(**A**) Venn diagram showing the overlap of DEGs between males and females at the PD, OD, and AD developmental stages. (**B**) STEM trend profiles based on union of DEGs across the three developmental stages. The horizontal axis represents the developmental stages (PD, OD, and AD), and the vertical axis represents the relative expression trend (normalized Log_2_Fold Change of Male vs. Female expression). The number in the top left corner of each profile indicates the profile ID, and the number in the bottom left corner indicates the number of genes within that profile. Colored profiles are significantly enriched (*p* < 0.05). (**C**) Heatmaps of genes in two significant profiles (Profile 7, Profile 8) across the three developmental stages, showing sex-specific expression patterns. (**D**-**G**) qPCR validation of RNA-seq expression levels for four DEGs (*ASB12*, *PTX4*, *IQCA1*, and *XPO4*). Each bar represents the mean ± SE for each sex across the PD, OD, and AD stages. (*n* = 4 biological replicates per sex at each developmental stage)

We compared sex-biased DEGs across the three developmental stages using Venn diagram analysis (Fig. 2A). 63 DEGs were shared among all stages, indicating persistent sex-biased expression throughout development, whereas the majority were stage-specific. Notably, the OD stage contained the largest set of unique DEGs (835 genes), highlighting a substantial transcriptional reprogramming concurrent with the onset of morphological dimorphism in FCR muscle. To explore temporal expression dynamics, we analyzed the union of DEGs with Short Time-series Expression Miner (STEM). Two significant expression profiles were identified (*p* < 0.05). Profile 7 (415 genes) showed a sharp increase in male-biased expression from PD to OD, followed by a decline in AD (Fig. 2B, Table S4). Profile 8 (177 genes) increased continuously from PD through AD. Heatmaps of these profiles revealed consistent male-biased upregulation at OD in both profiles, with Profile 8 sustaining high expression into AD (Fig. 2C, Table S4). These temporal patterns align with the developmental trajectory of FCR muscle mass dimorphism, suggesting that stage-specific activation of particular gene sets contributes to both the establishment and maintenance of sexually dimorphic muscle morphology.

### Validation of transcriptomic data by qRT-PCR

To verify the reliability of our RNA-seq analysis, we performed qRT-PCR validation on four candidate genes (*ASB12*, *PTX4*, *IQCA1*, and *XPO4*). These genes were selected to capture distinct temporal expression patterns and moderate fold changes, providing an unbiased assessment of dataset quality. Consistent with the RNA-seq results, *ASB12* and *PTX4* displayed markedly higher transcript levels in males than in females at the OD stage (Fig. 2D, E). In contrast, *IQCA1* and *XPO4* exhibited continuously elevated expression in males at both the OD and AD stages compared with females (Fig. 2F, G). The qPCR measurements for all selected genes demonstrated high concordance with the expression trends identified by transcriptome sequencing, further confirming the reliability of our transcriptomic dataset.

### GO and KEGG enrichment analyses

**Fig. 3:**
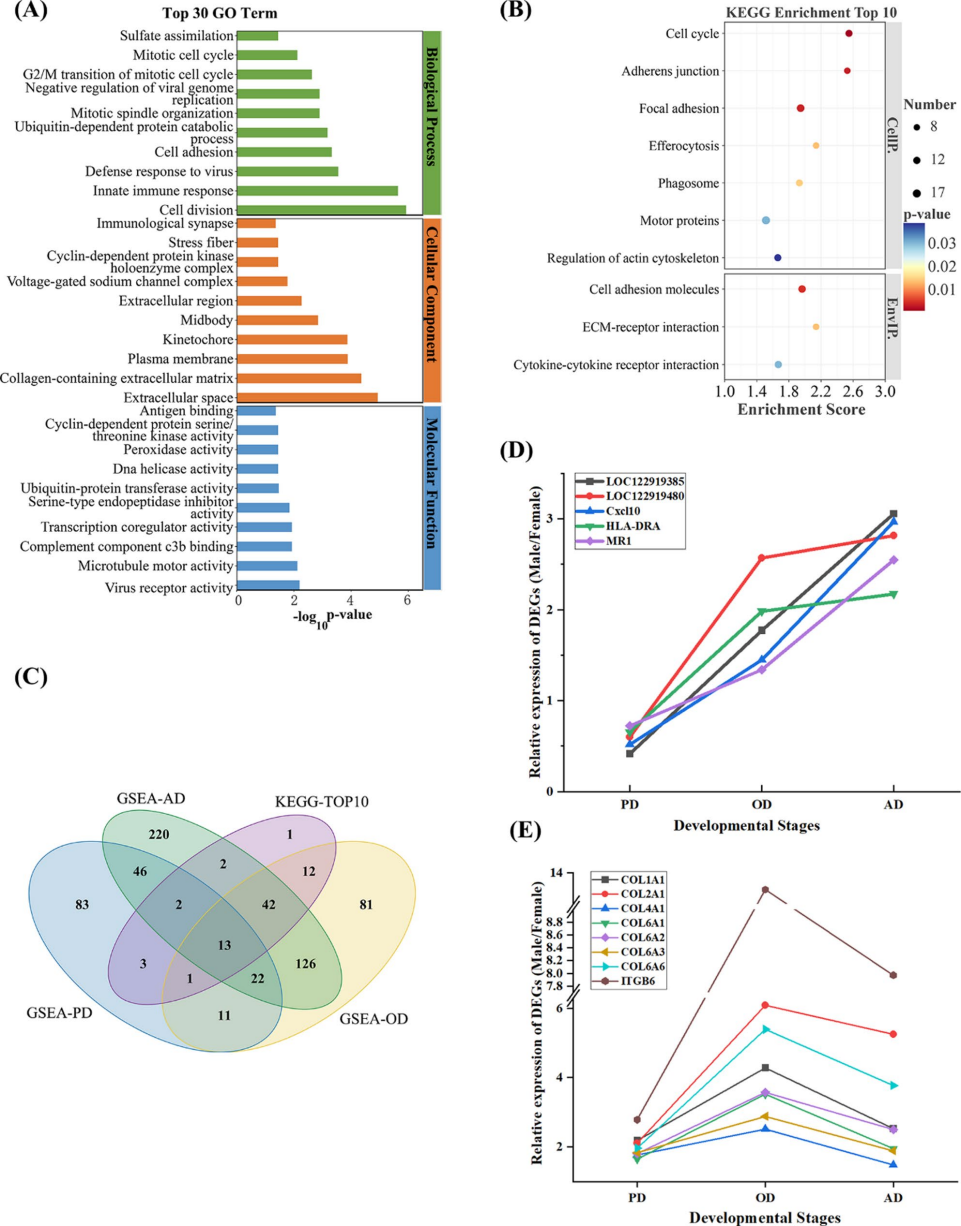
(**A**) Top 30 Gene Ontology (GO) terms for DEGs, classified into biological process, cellular component, and molecular function categories. (**B**) Top 10 KEGG pathways significantly enriched among DEGs. Pathways are ranked by enrichment score. Dot size represents the number of DEGs mapped to the pathway, and dot color indicates the p-value from the enrichment analysis. (**C**) Venn diagram showing overlap between KEGG‑Top 10 and GSEA results (PD, OD, AD stages). (**D**) Relative expression trends of five core DEGs across PD, OD, and AD stages. (**E**) Relative expression trends of eight ECM‑associated core DEGs across PD, OD, and AD stages

Based on the sex-biased DEGs identified by the STEM analysis, we performed GO and KEGG pathway enrichment analysis on the union set of 592 genes from Profile 7 and Profile 8. As shown in Fig. 3A, the GO analysis revealed the top 30 significant terms across the three major categories, including biological process (BP), cellular component (CC), and molecular function (MF). In the BP category, several terms were strongly associated with cell cycle progression and division, such as cell division, G2/M transition of mitotic cell cycle, and mitotic spindle organization, suggesting sex-specific differences in proliferative capacity of muscle tissues. In addition, cell adhesion was enriched, highlighting processes related to extracellular matrix (ECM) remodeling. In the CC category, DEGs were enriched in ECM- and cytoskeleton-associated structures, including extracellular space, collagen-containing extracellular matrix, plasma membrane, and stress fiber, indicating a notable role of ECM components and cytoskeletal organization in the structural differentiation between male and female FCR muscle. For MF, enriched functions included microtubule motor and cyclin-dependent kinase activities, consistent with cell cycle control, as well as serine-type endopeptidase inhibitor activity and ubiquitin-protein transferase activity, pointing to protein turnover and ECM remodeling processes. Collectively, these findings suggest that sexual dimorphism in FCR muscle may involve a coordinated program of cell proliferation and extensive structural remodeling, particularly of the ECM.

To explore upstream regulatory mechanisms that may contribute to these phenotypes, we performed KEGG pathway enrichment analysis (Fig. 3B, Table S5). Ten pathways were significantly enriched and clustered into cellular processes (CellP.) and environmental information processing (EnvIP.). In the CellP. category, the most significantly enriched pathway was cell cycle, consistent with GO results implicating proliferative regulation. Additional pathways related to cytoskeletal dynamics and cell adhesion–motor proteins, regulation of actin cytoskeleton, adherens junction, and focal adhesion–were also enriched. Examination of these pathways identified specific expression of growth factor signaling components, with *FGF10*, *FGF7*, and *FGFR1* significantly upregulated in males at the OD stage relative to females (Table S2; Table S5), suggesting enhanced growth signaling coupled to cell dynamics and structural remodeling. The EnvIP. category included cell adhesion molecules, ECM–receptor interaction, and cytokine–cytokine receptor interaction (Table S5). Notably, the focal adhesion pathway contained *IGF1*, a key regulator of skeletal muscle growth, which was significantly upregulated in males at the OD stage. In addition, the concurrent upregulation of *ITGB6* and *HRAS* within the ECM-receptor interaction pathway is consistent with more effective transduction of extracellular cues through downstream MAPK/ERK signaling. Overall, these results indicate that sexual dimorphism in FCR muscle is associated with coordinated activation of growth factor and ECM/integrin signaling pathways that may initiate and sustain the downstream cell proliferation and cytoskeletal remodeling programs characterized in our GO analysis.

### Integrative KEGG and GSEA analysis

We next performed KEGG-based Gene Set Enrichment Analysis (GSEA) independently for the PD, OD, and AD developmental stages using the full set of sex-biased DEGs identified at each stage (Table S6-S8), enabling verification of the functional enrichment patterns revealed by conventional KEGG analysis. Significantly enriched pathways (*p* < 0.05) from the three stages were combined into a union set and subsequently intersected with the top ten pathways from the conventional KEGG analysis to reduce potential false positives. We defined genes that were significantly enriched in both the standard KEGG pathway analysis and the stage-specific GSEA as key candidate genes. This intersection strategy ensures that the identified genes are robustly associated with the pathways of interest across different analytical methods. Using this criterion, we identified 13 candidate genes located in pathways supported by both methods (Fig. 3C). Among these, five genes (*Cxcl10*, *HLA-DRA*, *MR1*, *LOC122919385*, and *LOC122919480*) exhibited consistent male-biased expression from PD through AD (Fig. 3D). The remaining eight genes (*COL1A1*, *COL2A1*, *COL4A1*, *COL6A1*, *COL6A2*, *COL6A3*, *COL6A6*, and *ITGB6*) exhibited a pronounced upregulation in males from PD to OD, followed by a significant downregulation in AD (Fig. 3E). Notably, this core set comprised seven genes encoding structural collagen isoforms and one gene encoding an integrin receptor (*ITGB6*). The coordinated upregulation of these specific collagens and their transmembrane receptor (*ITGB6*) at the OD stage suggests the establishment of a functional mechanotransduction axis. This implies that collagen-integrin interactions likely drive the sexual dimorphism by linking extracellular matrix remodeling to the intracellular growth signaling pathways identified above.

### Protein-protein interaction (PPI) network

**Fig. 4:**
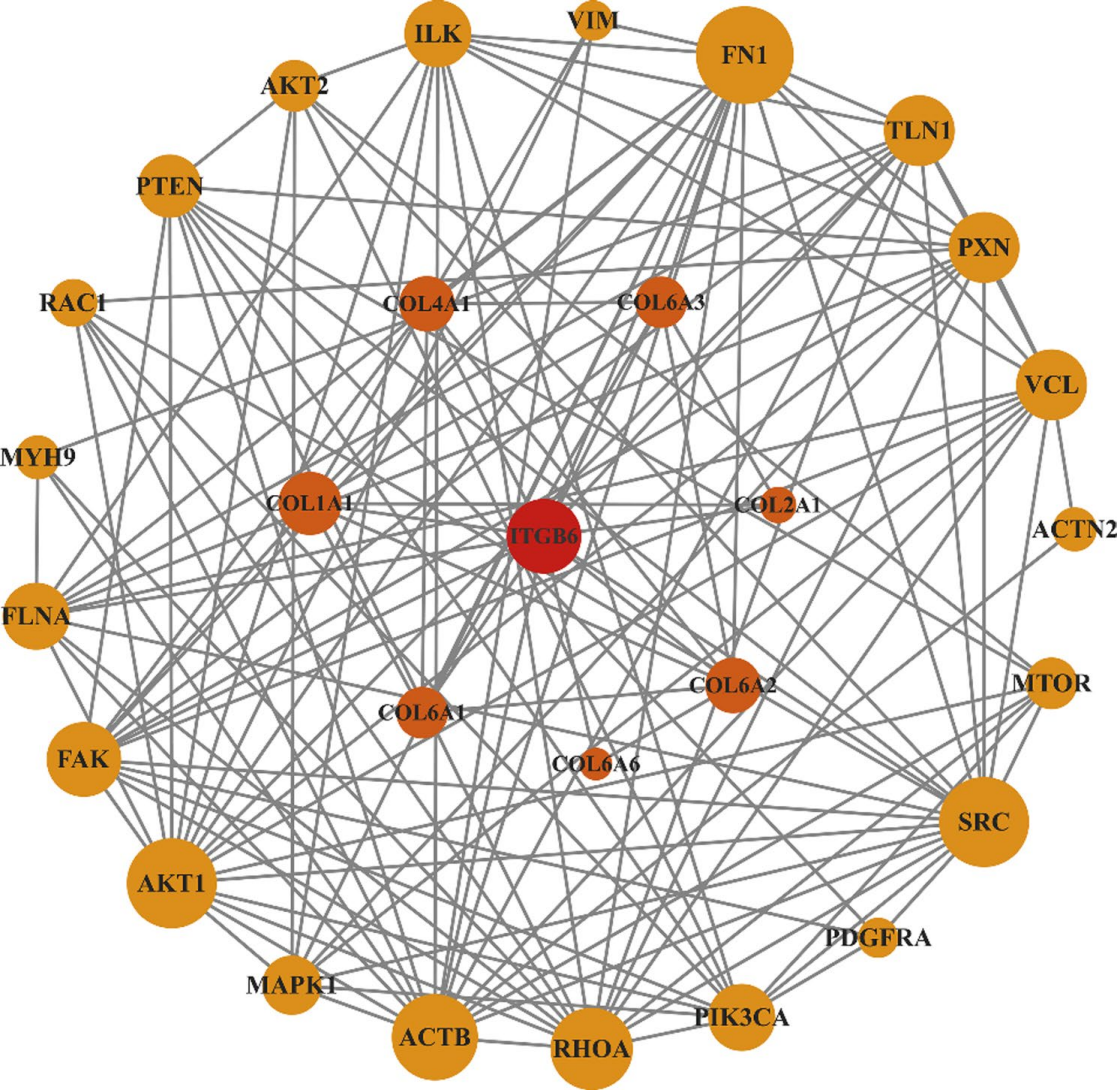
Protein-protein interaction (PPI) network (minimum interaction score > 0.700; STRING database) of eight ECM‑associated DEGs in the FCR muscle of *B. gargarizans*. Node size reflects connectivity degree

To further explore the functional relationships among the eight ECM-associated DEGs and proteins within their enriched pathways, a high-confidence protein-protein interaction (PPI) network (minimum interaction score > 0.700, STRING database) was constructed (Fig. 4). The network revealed that the seven collagen proteins (COL1A1, COL2A1, COL4A1, COL6A1, COL6A2, COL6A3, and COL6A6) form a tightly interconnected cluster. The integrin subunit ITGB6 emerged as the central hub, displaying direct associations with all seven collagens. Notably, ITGB6 was found to bridge this extracellular collagen matrix to two key groups of intracellular proteins: core components of the focal adhesion complex, including fibronectin (FN1), talin (TLN1), paxillin (PXN), and vinculin (VCL); and key kinases of the FAK signaling cascade, such as protein tyrosine kinase 2 (PTK2/FAK) and the SRC proto-oncogene (SRC). These results suggest that collagens and integrins cooperate to regulate both the structural frameworks and signaling pathways crucial for FCR muscle development, providing a molecular foundation for the observed male-specific hypertrophy.

## Discussion

By integrating morphological measurements with transcriptomic analyses across post-metamorphic development in *Bufo gargarizans*, we delineated the developmental onset and molecular basis of sexual dimorphism in the FCR muscle. Males exhibited significantly greater FCR muscle mass than females beginning at four months post‑metamorphosis (the onset of dimorphism, OD), well in advance of sexual maturity. This early onset indicates that muscle differentiation is initiated during the juvenile stage, significantly preceding functional reproductive activity, representing an adaptive preparation for subsequent amplexus behavior. Consistent with the morphological divergence, the OD stage displayed the highest number of sex‑biased DEGs, marking it as a critical window for sex‑specific transcriptional reprogramming. Notably, eight core DEGs (*COL1A1*, *COL2A1*, *COL4A1*, *COL6A1*, *COL6A2*, *COL6A3*, *COL6A6*, and *ITGB6*) were enriched in extracellular matrix (ECM) remodeling pathways and associated with downstream focal adhesion and FAK signaling. This coordinated upregulation indicates that collagen-integrin‑mediated ECM remodeling forms a key molecular substrate for the establishment of male‑specific FCR muscle hypertrophy.

Sexual dimorphism in the FCR muscle of anurans reflects sex-specific functional demands. The FCR contributes to coordinated wrist flexion and hand endorotation and serves as a critical component in locomotion [35]. In females, the muscle is thought to support routine biomechanical performance by acting as a linear stiffness spring that buffers landing impacts and stabilizes the wrist during substrate grasping [36]. In males, while maintaining these locomotor functions, the FCR must additionally sustain high-force isometric contractions during amplexus [37]. The early establishment of FCR muscle hypertrophy in male *B. gargarizans*, which occurs months before its functional role in amplexus, supports the interpretation of a programmed developmental strategy rather than a reactive response to sexual maturity. Such anticipatory development, where key morphological traits are completed well ahead of functional demand, is a widespread evolutionary solution for optimizing adult performance in predictable environments [38, 39]. For instance, *Spea multiplicata* can detect cues from nutrient-rich prey and respond by precociously enlarging their jaw musculature, thereby enhancing future feeding competitiveness [40]. Similarly, in oscine songbirds, the early crystallization of a complex song repertoire is crucial for ensuring readiness for future courtship displays [41, 42]. At the molecular level, such developmental strategies typically involve extensive transcriptional reprogramming that precedes overt morphological change [43, 44]. In *Drosophila melanogaster*, for example, transcriptional cascades induced by hormonal pulses occur well in advance of the morphological reconstruction of adult structures [45]. In male *B. gargarizans* FCR muscle, the OD stage is characterized by a marked increase in sex‑biased DEGs expression, indicating a pivotal phase in FCR muscle development. These transcriptional shifts may reflect an intrinsically timed genetic program that underlies the formation of the sexually dimorphic phenotype. The developmental sequence observed in the FCR muscle supports the view that major phenotypic divergence is accompanied by pre‑programmed shifts in gene regulatory networks [46, 47].

Functional enrichment analyses identified two primary regulatory processes underlying male FCR muscle hypertrophy: cell cycle–related pathways and ECM remodeling–related pathways. Given that skeletal muscle growth is traditionally attributed to an increase in the cross-sectional area of individual muscle fibers rather than an increase in the number of muscle fibers [48, 49], the enrichment of cell cycle–related pathways likely reflect the proliferation of muscle stem cells (satellite cells), rather than division of mature myofibers [50]. Indeed, the proliferation and subsequent fusion of satellite cells into existing myofibers are fundamental for muscle growth and adaptation [51]. Newly incorporated nuclei enlarge the myonuclear domain, thereby enhancing the transcriptional and translational capacities required for protein synthesis, driving fiber hypertrophy [52, 53]. Concurrently, enrichment of ECM-related pathways, including ECM-receptor interaction and cell adhesion, underscores ongoing dynamic remodeling of the tissue microenvironment. The ECM of vertebrate skeletal muscle is essential for maintaining mechanical integrity, yet its biological role extends well beyond serving as a passive scaffold [54]. It is now recognized as a dynamic signaling hub in myogenesis that integrates biochemical and mechanical cues to regulate satellite-cell adhesion, migration, proliferation, and differentiation, thereby coordinating muscle growth and regeneration [55, 56]. Moreover, ECM remodeling acts as a permissive switch for myogenesis, and its absence impairs satellite‑cell activation and differentiation even when growth factors are abundant [57]. Appropriate degradation, modification, and reassembly of ECM components modulate tissue mechanics and cell-matrix signaling, ultimately generating a microenvironmental niche conducive to satellite cell fate determination. This adaptive ECM remodeling is a highly complex and hierarchical biological process involving coordinated interactions among fibro-adipogenic progenitors (FAPs), immune cells, and myofibers [58]. It proceeds through distinct temporal phases: an early stage dominated by degradation and clearance, and a later stage characterized by new matrix synthesis [59]. In this study, expression of collagen and integrin genes was markedly upregulated in the FCR muscle at the OD stage in male *B. gargarizans*, suggesting extensive ECM reconstruction. Collectively, our results suggest that ECM remodeling may be a pivotal mechanism contributing to the pronounced FCR muscle hypertrophy observed in male *B. gargarizans*.

Our PPI network analysis suggests that seven collagen isoforms and the integrin subunit ITGB6 operate as a synergistic module to drive ECM restructuring, where collagens provide the structural substrate and ITGB6 acts as the anchor to transmit mechanical signals [60–63]. Integrin engagement is expected to promote recruitment and activation of focal adhesion kinase (PTK2/FAK), a key node that converts mechanical inputs into downstream biochemical responses [64–67]. Activated FAK can stimulate the anabolic PI3K-Akt-mTOR pathway by engaging upstream PI3K subunits and phosphorylating the mTOR repressor TSC2, thereby inactivating it and relieving inhibitory control of mTOR to trigger robust protein synthesis [68]. In addition, FAK modulates small GTPases (e.g., RHOA and RAC1) to exert a cytoskeletal‑remodeling function essential for precise myofibrillogenesis. RHOA activity is thought to be locally recruited at sarcomeric structures (e.g., the M‑band), where it guides the ordered assembly of actin and myosin filaments, ensuring efficient incorporation of newly synthesized proteins into functional contractile units [69–72]. Thus, FAK couples mass accumulation with structural optimization by concurrently activating anabolic synthesis and coordinating sarcomere organization. Therefore, ECM remodeling during the OD stage in male *B. gargarizans* may represent an adaptive mechanism that amplifies FAK‑pathway output, pre‑programming a structurally optimized muscle phenotype to meet later reproductive demands.

While we highlight the collagen-integrin-mediated ECM remodeling as a key effector of male-biased FCR hypertrophy, the upstream cues that initiate this process remain to be elucidated. Given the evolutionary conservation of musculoskeletal regulation, the sexually dimorphic response likely reflects the combined action of hormone signaling and intrinsic genetic factors [21, 73]. Our results parallel evidence from mammals showing that tissue-specific androgen sensitivity can strongly influence ECM remodeling in skeletal muscle. In mice, the highly androgen-sensitive perineal muscles serve as a canonical model for hormone-dependent growth and maintenance [74, 75]. Importantly, androgen sensitivity is not mediated solely by myofibers but also by mesenchymal progenitors (MPs). The androgen receptor (AR) in MPs upregulates collagen genes (e.g., *Col1a1*, *Col3a1*), supporting ECM organization and muscle integrity [76]. In myofibers, AR signaling further regulates integrin subunits (e.g., ITGB4, ITGB5) and structural proteins essential for force transmission and cytoskeletal integrity [77]. Similar to perineal muscles, the FCR is androgen dependent [78]. These findings imply that, at the OD stage, androgens may coordinate collagen and integrin expression in the FCR to couple ECM remodeling with intracellular growth pathways. Intrinsic genetic factors likely act in parallel to modulate this process. In *Xenopus laevis*, sexual differentiation is governed by antagonism between the conserved male factor *DMRT1* and its dominant-negative paralog *DM-W* [79]. They colocalize in somatic cells and compete for identical DNA-binding motifs, acting as a binary switch to direct tissue fate [80]. Beyond sex determination, genomic analyses in *Leptobrachium leishanense* indicate that male-specific nuptial spines are associated with lineage-specific expansion and spatially restricted expression of keratin gene families, accompanied by the local upregulation of androgen biosynthesis genes within the target tissue [81]. Therefore, the transcriptional profile characterized in our study likely represents the molecular convergence of hormonic and genetic inputs that drive ECM remodeling required for male-biased FCR growth.

## Conclusion

In summary, this study delineates a developmental molecular framework underlying the emergence of sexual dimorphism in the amplexus muscle of *B. gargarizans*. The results suggest that a stage‑specific surge in collagen and integrin gene expression at the OD stage mediates ECM remodeling, which in turn acts as a permissive switch for male‑specific muscle hypertrophy. This remodeling is predicted to initiate mechanotransductive signaling through downstream focal adhesion and FAK signaling network that coordinate protein synthesis, cytoskeletal organization, and structural development. Therefore, sexual dimorphism in the amplexus muscle of *B. gargarizans* is associated with an intrinsic, stage-specific program mediated by ECM‑mediated mechanical signaling. More broadly, our findings indicate that early ECM‑mediated signaling mechanistically links molecular differentiation to a sexually dimorphic phenotype established well before functional demand. This temporal coupling of ECM remodeling with adaptive gene regulation offers mechanistic insight into how sex‑specific musculoskeletal traits can evolve through developmental programming that anticipates adult reproductive requirements.

### Perspectives and significance

This study characterizes the onset of dimorphism (OD; 4 months post-metamorphosis) as a significant developmental window in *B. gargarizans*, during which collagen-integrin-mediated ECM remodeling promotes the persistent sexual dimorphism of the FCR muscle prior to sexual maturation. By integrating morphological timelines with transcriptomic data, we reveal coordinated male‑biased upregulation of seven collagen isoforms and ITGB6 at OD, linking ECM remodeling to downstream focal adhesion and FAK signaling. Our work provides a developmental genetic framework for sex‑specific muscle growth in anurans, suggesting how developmental molecular events drive adult performance traits. Future comparative and functional studies targeting collagen-integrin interactions could test the generality of this mechanism across vertebrates and determine how ecological and endocrine factors shape sexually dimorphic musculoskeletal adaptations.

## Supplementary Information

Below is the link to the electronic supplementary material.

Supplementary Material 1

Supplementary Material 2

Supplementary Material 3

Supplementary Material 4

Supplementary Material 5

Supplementary Material 6

Supplementary Material 7

Supplementary Material 8

## Data Availability

The raw RNA-seq data are available at the Genome Sequence Archive (GSA) under the accession number CRA033326 ( https://ngdc.cncb.ac.cn/gsa/s/5tO1076Z ).
